# Short-term effects of ambient temperature on the risk of preeclampsia in Nanjing, China: a time-series analysis

**DOI:** 10.1186/s12884-022-04859-w

**Published:** 2022-07-04

**Authors:** Tingting Zhao, Wei Long, Peng Lu

**Affiliations:** 1 Women’s Hospital of Nanjing Medical University, Nanjing Maternity and Child Health Care Hospital, Nanjing, China; 2 Jiangsu Climate Centre, Nanjing, China

**Keywords:** Preeclampsia, Daily temperature, DLNM (distributed lag nonlinear model), Short-term effect, Lag effects

## Abstract

**Objectives:**

Previous studies on the association between temperature and preeclampsia mainly considered temperature on a monthly or seasonal time scale. The objective of this study was to assess the preeclampsia risk associated with short-term temperature exposure using daily data.

**Study design:**

Daily preeclampsia hospitalization data, daily meteorological data and daily air pollutant data from Nanjing were collected from 2016 to 2017. Both the T test and distributed lag nonlinear model (DLNM) were applied to assess the short-term effect of temperature on preeclampsia risk. Three kinds of daily temperature, including the daily mean temperature, daily minimum temperature and daily maximum temperature, were analysed.

**Results:**

When the daily number of preeclampsia hospital admissions was divided into two subgroups based on temperature, it was significantly larger on cold days than on hot days. Regarding the mean temperature, a very low level of mean temperature (4.5 °C, lag = 0–20) and a low level of mean temperature (9.1 °C, lag = 0–20) increased the cumulative relative risk of preeclampsia by more than 60%. At the same time, a very high level of mean temperature (28.7 °C, lags = 0–10, 0–15, 0–20) and a high level of mean temperature (24.1 °C, lags = 0–10, 0–15) decreased the cumulative relative risk of preeclampsia by more than 35%. At a minimum temperature, a very low level of minimum temperature (0.9 °C, lag 0–5) and a low level of minimum temperature (5.6 °C, lag 0–5) increased the cumulative relative risk of preeclampsia by more than 55%. At the same time, a high level of mean temperature (20.9 °C, lags = 0, 0–5) decreased the cumulative relative risk of preeclampsia by more than 20%. The maximum temperature result was similar to the mean temperature result.

**Conclusions:**

Both direct and lag effects of low temperature on preeclampsia were demonstrated to be significant risk factors. These results could be used to help pregnant women and the government reduce preeclampsia risk.

**Supplementary Information:**

The online version contains supplementary material available at 10.1186/s12884-022-04859-w.

## Introduction

Preeclampsia is a pregnancy-specific syndrome that affects 3–5% of pregnant women and is traditionally diagnosed when a pregnant woman presents with increased blood pressure and proteinuria [1, 2]. Recently, the association between temperature and preeclampsia risk was evaluated. Most previous studies analysed seasonal or monthly temperature data [3–5]. They found that preeclampsia risk was increased in women who conceived during the warm months and delivered during the cold months [3–5]. There was only one paper that studied the association between maximum temperature and preeclampsia risk considering daily data [6]. Basu [7] pointed out that preeclampsia risk should be explored considering both short-term and long-term temperature exposure windows. In the current study, to assess the preeclampsia risk associated with short-term temperature exposure windows, daily temperature data and daily hospital preeclampsia admission data were analysed. To effectively assess the short-term effect, we estimated preeclampsia based on the daily number of preeclampsia hospital admissions, not conception time or delivery time. This was different from previous studies [3–5]. Basu [7] also pointed out that temperature metrics including the mean, minimum and maximum temperatures should be considered. In this study, three kinds of daily temperature were studied to determine whether different kinds of daily temperature had different effects on preeclampsia risk.

## Data and methods

### Data collection

We obtained preeclampsia hospital admission data from the electronic medical records of Nanjing Maternity and Child Health Care Hospital from 2016–2017. We identified preeclampsia admissions using the International Classification of Diseases, 10th Revision (ICD-10) codes O13 and O14; O13 indicated mild preeclampsia and O14 indicated severe preeclampsia. The data used in this study were collected without any individual identifiers. This study was approved by the Institutional Review Board of Nanjing Maternity and Child Health Care Hospital, and the methods were carried out in accordance with the approved guidelines.

We obtained the daily mean temperature, daily minimum temperature, daily maximum temperature and daily relative humidity in Nanjing during 2016–2017 from the Jiangsu Meteorological Bureau.

We obtained the daily SO_2_, NO_2_, CO, O_3_, PM_10_, and PM_2.5_ in Nanjing during 2016–2017 from the China National Environmental Monitoring Centre.

### Statistical analysis

The distributed lag nonlinear model (DLNM) is a model used to describe potentially nonlinear and delayed dependencies [8].

$$\mathrm{Log}\lbrack\mathrm E({\mathrm Y}_{\mathrm t})\rbrack=\;\mathrm a\;+\;\mathrm{cb}({\mathrm{temp}}_{\mathrm t})\;+\;\mathrm{ns}(\mathrm{RH},\;3)\;+\;\mathrm{ns}({\mathrm{SO}}_2,3)\;+\;\mathrm{ns}({\mathrm{NO}}_2,3)\;+\;\mathrm{ns}(\mathrm{CO},3)\;+\;\mathrm{ns}({\mathrm O}_3,3)\;+\;\mathrm{ns}({\mathrm{PM}}_{10},3)\;+\;\mathrm{ns}({\mathrm{PM}}_{2.5},3)$$

where Y_t_ is the daily number of preeclampsia hospital admissions on day t; a is the intercept; and cb is the cross-basis function. A natural cubic spline was used to model the nonlinear association between temperature and the number of preeclampsia hospital admissions. Moreover, the natural cubic spline method was used to fit relative humidity (RH), SO_2_, NO_2_, CO, O_3_, PM_10_ and PM_2.5._

The analyses were performed with the software R, version 4.0.0. The package DLNM [9] was used to specify and interpret the results in this study.

In this study, we focused on the short term effects of ambient temperature on the risk of preeclampsia, so the lag effects on the day after 30 days were not considered. After calculation, the maximum lag of the mean temperature and maximum temperature was 30 days, and the maximum lag of the minimum temperature was 6 days. The details are provided in Supplemental Material A [10].

The degrees of freedom were 3 when using natural cubic spline method to fit relative humidity (RH), SO_2_, NO_2_, CO, O_3_, PM_10_ and PM_2.5_ based on BIC method in Table S3.

### Sensitivity analysis

Sensitivity analysis was conducted by varying df (degrees of freedom) to examine the robustness of the results. There were no large differences between the different df values when using natural cubic spline method to fit relative humidity (RH), SO_2_, NO_2_, CO, O_3_, PM_10_ and PM_2.5_. The details are shown in Table S4 and Table S5.

## Results

During 2016 and 2017, there were a total of 1213 recorded preeclampsia hospital admissions at Nanjing Maternity and Child Health Care Hospital. Table 1 summarizes the distributions of daily preeclampsia hospital admissions according to different temperature ranges. For the daily mean temperature, when the mean temperature was between 0 and 9.9 °C, the daily number of preeclampsia hospital admissions was the highest, with a value of 1.81; when the temperature was higher than or equal to 30 °C, the daily number of preeclampsia hospital admissions was the lowest, with a value of 1.39. For the daily minimum temperature, when the minimum temperature was between 0 and 9.9 °C, the daily number of preeclampsia hospital admissions was the highest, with a value of 1.90. There was only one day in which the minimum temperature was higher than or equal to 30 °C; the daily minimum temperature on this day was 30.3 °C. When the minimum temperature was between 20 °C and 29.9 °C, the daily number of preeclampsia hospital admissions was the second lowest, with a value of 1.47. For the daily maximum temperature, when the maximum temperature was between 10 °C and 19.9 °C, the number of daily preeclampsia hospital admissions was the highest, with a value of 1.87. There was only one day in which the maximum temperature was less than 0 °C; the daily maximum temperature on this day was -3.0 °C. When the maximum temperature was higher than or equal to 30 °C, the daily number of preeclampsia hospitalizations was the second lowest, with a value of 1.50.

**Table 1:** Number of preeclampsia hospital admissions according to daily temperature, including mean, minimum and maximum temperatures, in Nanjing during 2016–2017

	Daily mean temperature			Daily minimum temperature			Daily maximum temperature	
Temperature (°C)	Number of Preeclampsia admissions	Preeclampsia admissions per day (95% confidence interval)	Number of Preeclampsia admissions		Preeclampsia admissions per day (95% confidence interval)	Number of Preeclampsia admissions		Preeclampsia admissions per day (95% confidence interval)
< 0	12	1.71 (0.69–2.74)	102		1.79 (1.41–2.17)	0		0
0 to 9.9	355	1.81 (1.58–2.04)	412		1.90 (1.68–2.12)	182		1.82 (1.51–2.13)
10 to 19.9	378	1.73 (1.53–1.94)	393		1.58 (1.40–1.75)	423		1.87 (1.66–2.09)
20 to 29.9	393	1.54 (1.36–1.71)	305		1.47 (1.28–1.67)	376		1.51 (1.33–1.69)
≥ 30	75	1.39 (1.06–1.72)	1		1.00	232		1.50 (1.29–1.71)
Total	1213	1.66 (1.55–1.77)	1213		1.66 (1.55–1.77)	1213		1.66 (1.55–1.77)

As shown in Table 2, the preeclampsia hospital admission records were divided into two subgroups, and the median was used as the grouping threshold. The daily mean temperature threshold was 17.2 °C, the daily minimum temperature threshold was 14.4 °C, and the daily maximum temperature threshold was 21.6 °C. The daily number of preeclampsia hospital admissions was significantly higher in the subgroup with a daily mean temperature less than 17.2 °C than in the subgroup with a daily mean temperature higher than or equal to17.2 °C (1.82 vs. 1.48, *P* = 0.002). Similarly, the daily number of preeclampsia hospital admissions was significantly higher in the subgroup with a daily minimum temperature less than 14.4 °C than in the subgroup with a daily minimum temperature higher than or equal to 14.4 °C (1.82 vs. 1.50, *P* = 0.004). Moreover, the daily number of preeclampsia hospital admissions was significantly higher in the subgroup with a daily maximum temperature less than 21.6 °C than in the subgroup with a daily maximum temperature higher than or equal to 21.6 °C (1.82 vs. 1.50, *P* = 0.003). Considering 17.2 °C, 14.4 °C, and 21.6 °C as temperature thresholds corresponding to the daily mean temperature, daily minimum temperature and daily maximum temperature, there were significant differences between the subgroups of preeclampsia hospital admission numbers.

**Table 2:** Difference between the two subgroups according to temperature threshold (the daily mean temperature threshold was 17.2 °C, the daily minimum temperature threshold was 14.4 °C, and the daily maximum temperature threshold was 21.6 °C)

Mean temperature (days)	Preeclampsia admissions per day (95% confidence interval)	Minimum temperature (days)	Preeclampsia admissions per day(95% confidence interval)	Maximum temperature (days)	Preeclampsia admissions per day(95% confidence interval)
> = 17.2 (365)	1.48 (1.34–1.63)	> = 14.4 (364)	1.50 (1.36–1.64)	> = 21.6(365)	1.50 (1.36–1.64)
< 17.2(366)	1.82 (1.66–1.99)	< 14.4(367)	1.82 (1.65–1.98)	< 21.6(366)	1.82 (1.66–1.99)
t	-3.11	t	-2.87	t	-2.96
p	0.002	p	0.004	p	0.003

**Fig. 1:**
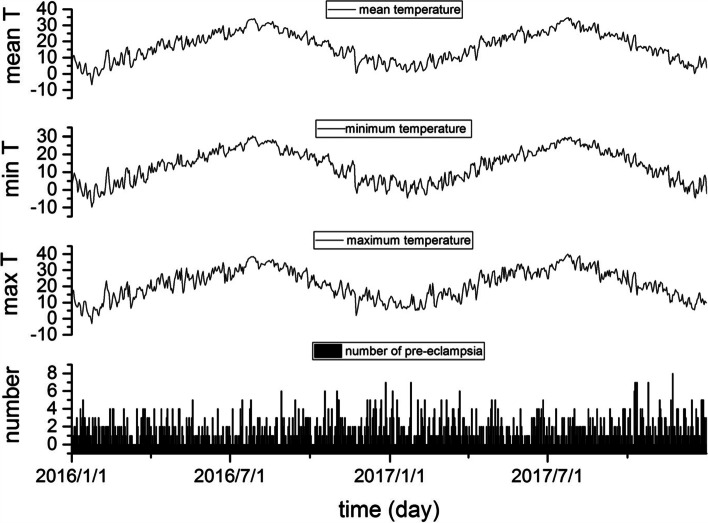
Daily temperature and the daily number of preeclampsia hospital admissions time series (from top to bottom: the daily mean temperature, daily minimum temperature, daily maximum temperature and daily number of preeclampsia hospital admissions)

As shown in Fig. 1, the Pearson correlation coefficients among the three kinds of daily temperature were high, at 0.93 or higher (*P* < 0.001 for all). Moreover, the daily number of preeclampsia hospital admissions time series also indicated a weak correlation with the three daily temperature time series. The Pearson correlation coefficient between the daily number of preeclampsia hospital admissions time series and the daily mean temperature time series was -0.103 (*P* = 0.005); the Pearson correlation coefficient between the daily number of preeclampsia hospital admission time series and the daily minimum temperature time series was -0.106 (*P* = 0.004); and the Pearson correlation coefficient between the daily number of preeclampsia hospital admissions time series and the daily maximum temperature time series was -0.096 (*P* = 0.01).

To study the short-term effects of daily temperature on the number of preeclampsia admissions, the DLNM model was used. The median daily temperature was set as the reference temperature.

**Fig. 2:**
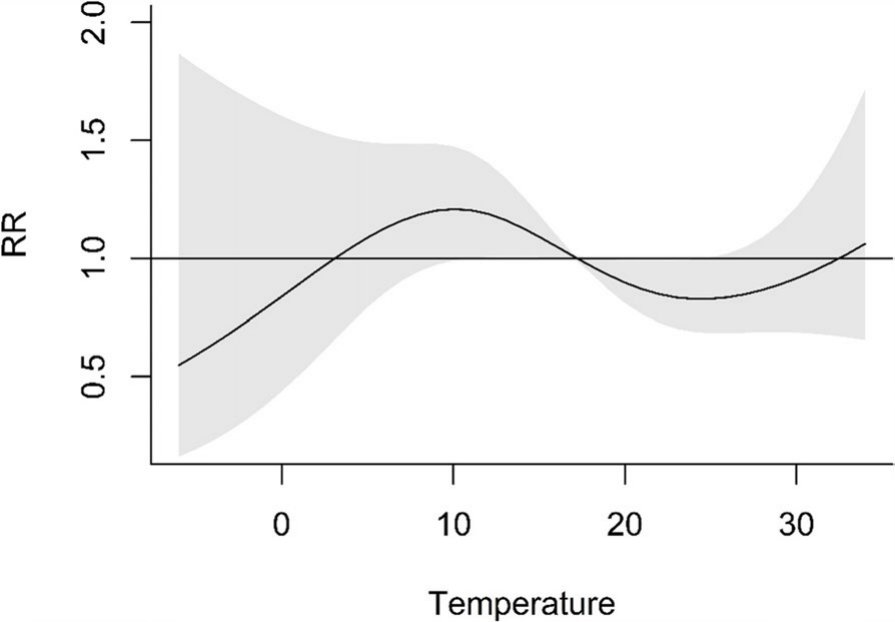
Cumulative exposure–response associations of the daily mean temperature and number of preeclampsia hospital admissions in Nanjing, China, 2016–2017

In Fig. 2, the overall cumulative exposure–response association between the daily number of preeclampsia hospital admissions and the daily mean temperature across a 30-day lag was shown. The reference temperature was 17.2 °C (median of the mean temperature). When the mean temperature was lower than the reference temperature, the relative risk was lowest, at 0.52 (95%CI: 0.14–1.90), when the daily mean temperature was -6.7 °C; then, the relative risk curve increased from a value less than 1.0 to a value higher than 1.0. When the daily mean temperature was 10.1 °C, the relative risk peaked at 1.21 (95%CI: 0.99–1.47); when the mean temperature was higher than 10.1 °C, the relative risk decreased to 1.0 with the mean temperature as the reference temperature. At the same time, when the mean temperature was higher than the reference temperature, the relative risk curve decreased, and when the daily mean temperature was 24.4 °C, the relative risk was the local minimum at 0.83 (95%CI: 0.69–1.00); the relative risk curve increased slowly thereafter. When the mean temperature was higher than or equal to 32.5 °C, the relative risk curve was more than 1.0.

The single and cumulative lag effects of the mean temperature on the number of preeclampsia hospital admissions, with 17.2 °C as reference, were shown in Table 3. We defined 4.5 °C (the 10th percentile of mean temperature) as very low level of mean temperature; 9.1 °C (the 25th percentile of mean temperature) as low level of mean temperature; 24.1 °C (the 75th percentile of mean temperature) as high level of mean temperature; and 28.7 °C (the 90th percentile of mean temperature) as very high level of mean temperature. There were no significant single effects for most of the mean temperature, only a single effect on a lag of 5 days (RR = 0.97, 95%CI: 0.93–1.00) on a high level of mean temperature was significant. At a very low level of mean temperature, only the cumulative effect on a lag of 0–20 days (RR = 1.73, 95%CI: 1.06–2.83) was significant. At a low level of mean temperature, the cumulative effect changed the risk of preeclampsia from a lag of 0–10 days (RR = 1.49, 95%CI: 1.07–2.09) and lasted until a lag of 0–25 days (RR = 1.54, 95%CI: 1.09–2.18). The greatest cumulative effect was observed on a lag of 0–20 days (RR = 1.62, 95%CI: 1.14–2.29). At a high level of mean temperature, the cumulative effect changed the risk of preeclampsia from a lag of 0–5 days (RR = 0.69, 95%CI: 0.51–0.93) and lasted until a lag 0–30 of days (RR = 0.83, 95%CI: 0.69–1.00). The lowest cumulative effect was observed on a lag of 0–10 days (RR = 0.64, 95%CI: 0.48–0.86). At a very high level of mean temperature, the cumulative effect changed the risk of preeclampsia from a lag of 0–10 days (RR = 0.60, 95%CI: 0.39–0.90) and lasted until a lag of 0–20 days (RR = 0.64, 95%CI: 0.43–0.97). The lowest cumulative effect was observed on a lag of 0–15 days (RR = 0.59, 95%CI: 0.39–0.89).

**Table 3:** The single and cumulative effects estimated for different mean temperatures at different lag days with the reference of 17.2 °C (median)

Single lag days	4.5 °C (the 10th percentile) RR (95% confidence interval)	9.1 °C (the 25th percentile) RR (95% confidence interval)	24.1 °C (the 75th percentile) RR (95% confidence interval)	28.7 °C (the 90th percentile) RR (95% confidence interval)	Multiple lag days	4.5 °C (the 10th percentile) RR (95% confidence interval)	9.1 °C (the 25th percentile) RR (95% confidence interval)	24.1 °C (the 75th percentile) RR (95% confidence interval)	28.7 °C (the 90th percentile) RR (95% confidence interval)
0	1.02 (0.88–1.18)	1.08 (0.96–1.20)	0.91 (0.82–1.01)	0.92 (0.78–1.07)	0	1.02 (0.88–1.18)	1.08 (0.96–1.20)	0.91 (0.82–1.01)	0.92 (0.78–1.07)
5	1.03 (0.98–1.08)	1.03 (0.99–1.07)	0.97 (0.93–1.00)*	0.96 (0.91–1.00)	0–5	1.15 (0.73–1.79)	1.35 (0.98–1.86)	0.69 (0.51–0.93)*	0.68 (0.44–1.05)
10	1.03 (0.98–1.10)	1.01 (0.97–1.06)	0.99 (0.95–1.04)	0.99 (0.93–1.05)	0–10	1.34 (0.82–2.18)	1.49 (1.07–2.09)*	0.64 (0.48–0.86)*	0.60 (0.39–0.90)*
15	1.03 (0.98–1.08)	1.01 (0.97–1.05)	1.01 (0.97–1.04)	1.01 (0.96–1.06)	0–15	1.58 (0.95–2.60)	1.57 (1.11–2.23)*	0.65 (0.48–0.87)*	0.59 (0.39–0.89)*
20	1.01 (0.95–1.07)	1.00 (0.96–1.05)	1.01 (0.97–1.05)	1.02 (0.96–1.09)	0–20	1.73 (1.06–2.83)*	1.62 (1.14–2.29)*	0.68 (0.50–0.91)*	0.64 (0.43–0.97)*
25	0.96 (0.92–1.01)	0.98 (0.95–1.01)	1.02 (0.99–1.05)	1.03 (0.99–1.08)	0–25	1.60 (0.99–2.58)	1.54 (1.09–2.18)*	0.72 (0.53–0.99)*	0.74 (0.48–1.15)
30	0.89 (0.78–1.02)	0.93 (0.83–1.04)	1.04 (0.94–1.15)	1.04 (0.89–1.20)	0–30	1.07 (0.76–1.50)	1.20 (0.97–1.48)	0.83 (0.69–1.00)*	0.88 (0.69–1.13)

* *p*<0.05

**Fig. 3:**
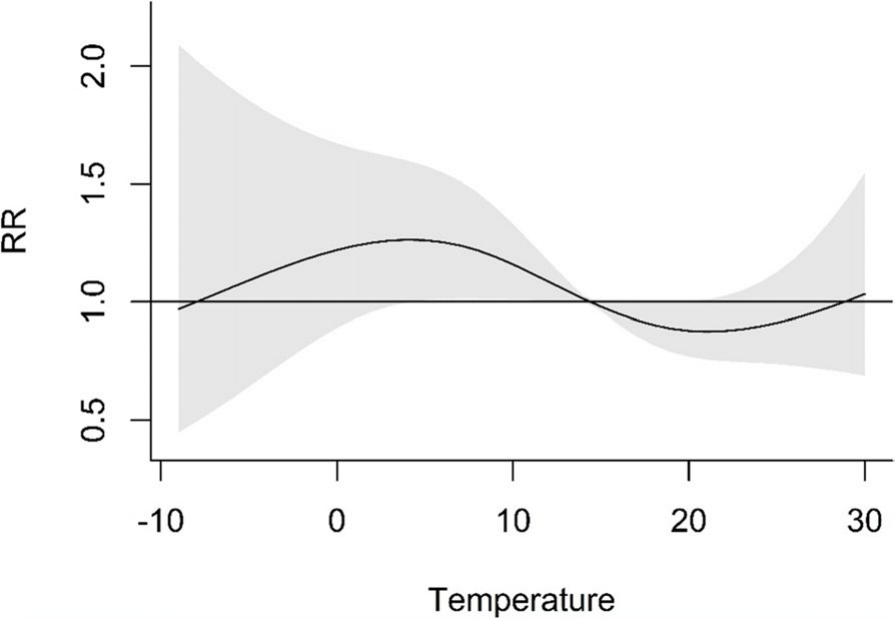
Cumulative exposure–response associations of the daily minimum temperature and number of preeclampsia hospital admissions in Nanjing, China, 2016–2017

In Fig. 3, the overall cumulative exposure–response association between the daily number of preeclampsia hospital admissions and daily minimum temperature across a 6-day lag was shown. The reference temperature was 14.4 °C. The relative risk curve in Fig. 3 was similar to the curve in Fig. 2. The relative risk was highest, at 1.26 (95%CI: 1.00–1.60), when the daily minimum temperature was 4.1 °C. The relative risk was lowest, at 0.88 (95%CI: 0.76–1.01), when the daily minimum temperature was 21.0 °C.

With the reference of 14.4 °C, the single and cumulative lag effects of the minimum temperature on the number of preeclampsia hospital admissions were shown in Table 4. We defined 0.9 °C (the 10th percentile of minimum temperature) as very low level of minimum temperature; 5.6 °C (the 25th percentile of minimum temperature) as low level of minimum temperature; 20.9 °C (the 75th percentile of minimum temperature) as high level of minimum temperature; 25.7 °C (the 90th percentile of minimum temperature) as very high level of minimum temperature. At a very low level of minimum temperature, there was a single effect on a lag of 4 days (RR = 1.27, 95%CI: 1.01–1.59) and a cumulative effect on a lag of 0–5 days (RR = 1.65, 95%CI: 1.07–2.54). At a low level of minimum temperature, the single effect decreased the risk of preeclampsia from a lag of 4 days (RR = 1.21, 95%CI: 1.00–1.46) to a lag of 5 days (RR = 1.18, 95%CI: 1.00–1.40), and the cumulative effect decreased the risk of preeclampsia from a lag of 0–5 days (RR = 1.55, 95%CI: 1.10–2.19) to a lag of 0–6 days (RR = 1.26, 95%CI: 1.01–1.56). At a high level of minimum temperature, the single effect on lags of 0 days (RR = 0.76, 95%CI: 0.59–0.98) and 5 days (RR = 0.87, 95%CI: 0.76–0.99) was statistically significance, while the cumulative effect on a lag of 0–5 days (RR = 0.76, 95%CI: 0.58–0.99) was statistically significance. At a very high level of minimum temperature, there were no significant single or cumulative effects.

**Table 4:** The single and cumulative effects estimated for different minimum temperatures at different lag days with a reference of 14.4 °C (median)

Single lag days	0.9 °C (the 10th percentile) RR(95% confidence interval)	5.6 °C (the 25th percentile) RR (95% confidence interval)	20.9 °C (the 75th percentile) RR (95% confidence interval)	25.7 °C (the 90th percentile) RR (95% confidence interval)	Multiple lag days	0.9 °C (the 10th percentile) RR (95% confidence interval)	5.6 °C (the 25th percentile) RR (95% confidence interval)	20.9 °C (the 75th percentile) RR (95% confidence interval)	25.7 °C (the 90th percentile) RR (95% confidence interval)
0	1.34 (0.90–1.99)	1.31 (0.95–1.81)	0.76 (0.59–0.98)*	0.65 (0.39–1.08)	0	1.34 (0.90–1.99)	1.31 (0.95–1.81)	0.76 (0.59–0.98)*	0.65 (0.39–1.08)
1	0.87 (0.71–1.08)	0.90 (0.76–1.07)	1.11 (0.97–1.28)	1.22 (0.92–1.62)	0–1	1.17 (0.79–1.73)	1.19 (0.86–1.63)	0.85 (0.66–1.09)	0.79 (0.48–1.30)
2	0.87 (0.68–1.10)	0.89 (0.73–1.08)	1.16 (0.99–1.36)	1.32 (0.96–1.83)	0–2	1.01 (0.68–1.51)	1.05 (0.76–1.45)	0.99 (0.76–1.27)	1.05 (0.63–1.74)
3	1.05 (0.89–1.24)	1.04 (0.90–1.19)	1.02 (0.91–1.14)	1.07 (0.85–1.36)	0–3	1.07 (0.71–1.60)	1.09 (0.78–1.51)	1.00 (0.77–1.30)	1.13 (0.68–1.86)
4	1.27 (1.01–1.59)*	1.21 (1.00–1.46)*	0.87 (0.75–1.02)	0.84 (0.61–1.15)	0–4	1.35 (0.90–2.03)	1.32 (0.95–1.82)	0.88 (0.68–1.13)	0.94 (0.58–1.54)
5	1.22 (0.99–1.49)	1.18 (1.00–1.40)*	0.87 (0.76–0.99)*	0.80 (0.61–1.06)	0–5	1.65 (1.07–2.54)*	1.55 (1.10–2.19)*	0.76 (0.58–0.99)*	0.76 (0.46–1.26)
6	0.75 (0.53–1.06)	0.81 (0.61–1.08)	1.15 (0.91–1.46)	1.22 (0.76–1.98)	0–6	1.24 (0.93–1.65)	1.26 (1.01–1.56)*	0.88 (0.76–1.01)	0.92 (0.73–1.17)

* *p*<0.05

**Fig. 4:**
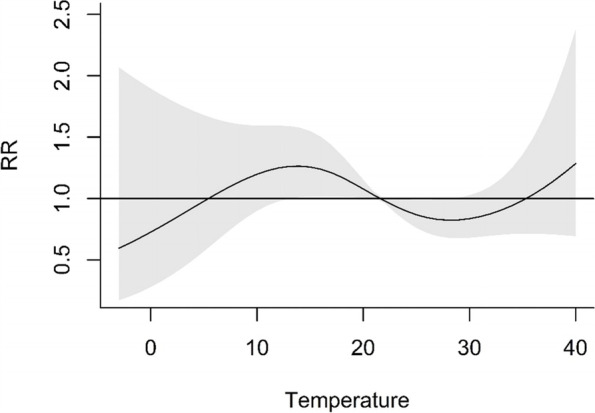
Cumulative exposure–response associations of the daily maximum temperature and the number of preeclampsia hospital admissions in Nanjing, China, 2016–2017

In Fig. 4, the overall cumulative exposure–response association between the daily number of preeclampsia admissions and the daily maximum temperature across a 7-day lag was shown. The reference temperature was 21.6 °C. The relative risk curve in Fig. 4 was also similar to the curve in Fig. 2. The relative risk was lowest, at 0.59 (95%CI: 0.17–2.07), when the daily maximum temperature was -3.0 °C. The relative risk was highest, at 1.28 (95%CI: 0.69–2.38), when the daily maximum temperature was 40.0 °C.

With the reference of 21.6 °C, the single and cumulative lag effects of the maximum temperature on the number of preeclampsia hospital admissions were shown in Table 5. We defined 8.8 °C (the 10th percentile of maximum temperature) as very low level of maximum temperature; 14.2 °C (the 25th percentile of maximum temperature) as low level of maximum temperature; 29.0 °C (the 75th percentile of maximum temperature) as high level of maximum temperature; 33.2 °C (the 90th percentile of maximum temperature) as very high level of maximum temperature. At a very low level of maximum temperature, the cumulative effect changed the risk of preeclampsia from a lag of 0–15 days (RR = 1.62, 95%CI: 1.04–2.51) and lasted until a lag of 0–25 days (RR = 1.58, 95%CI: 1.02–2.45). The greatest cumulative effect was observed on a lag of 0–20 days (RR = 1.72, 95%CI: 1.10–2.67). At a low level of maximum temperature, the single effect on a lag of 5 days (RR = 1.04, 95%CI: 1.01–1.07) was significant, and the cumulative effect changed the risk of preeclampsia from a lag of 0–5 days (RR = 1.32, 95%CI: 1.00–1.74) and lasted until a lag of 0–30 days (RR = 1.26, 95%CI: 1.01–1.57). The greatest cumulative effect on lag 0–20 days (RR = 1.63, 95%CI: 1.19–2.22). At a high level of maximum temperature, the single effect on a lag of 5 days (RR = 0.96, 95%CI: 0.93–0.99) was significant, and the cumulative effect changed the risk of preeclampsia from a lag of 0–10 days (RR = 0.65, 95%CI: 0.48–0.87) and lasted until a lag of 0–25 days (RR = 0.72, 95%CI: 0.54–0.97). The lowest cumulative effect was observed on a lag of 0–15 days (RR = 0.62, 95%CI: 0.45–0.83). At a very high level of maximum temperature, the single effect on a lag of 5 days (RR = 0.96, 95%CI: 0.92–1.00) was significant, and the cumulative effect changed the risk of preeclampsia from a lag of 0–10 days (RR = 0.64, 95%CI: 0.43–0.95) and lasted until a lag of 0–20 days (RR = 0.64, 95%CI: 0.44–0.94). The lowest cumulative effect was observed on a lag of 0–15 days (RR = 0.60, 95%CI: 0.41–0.88).

**Table 5:** The single and cumulative effects estimated for different maximum temperatures at different lag days with the reference of 21.6 °C (median)

Single lag days	8.8 °C (the 10th percentile) RR (95% confidence interval)	14.2 °C (the 25th percentile) RR (95% confidence interval)	29.0 °C (the 75th percentile) RR (95% confidence interval)	33.2 °C (the 90th percentile) RR (95% confidence interval)	Multiple lag days	8.8 °C (the 10th percentile) RR (95% confidence interval)	14.2 °C (the 25th percentile) RR (95% confidence interval)	29.0 °C (the 75th percentile) RR (95% confidence interval)	33.2 °C (the 90th percentile) RR (95% confidence interval)
0	1.03 (0.91–1.16)	1.06 (0.96–1.16)	0.95 (0.86–1.05)	0.96 (0.84–1.11)	0	1.03 (0.91–1.16)	1.06 (0.96–1.16)	0.95 (0.86–1.05)	0.96 (0.84–1.11)
5	1.03 (0.99–1.08)	1.04 (1.01–1.07)*	0.96 (0.93–0.99)*	0.96 (0.92–1.00)*	0–5	1.20 (0.83–1.75)	1.32 (1.00–1.74)*	0.75 (0.56–1.00)	0.77 (0.52–1.16)
10	1.03 (0.98–1.08)	1.02 (0.98–1.06)	0.98 (0.94–1.02)	0.97 (0.92–1.02)	0–10	1.41 (0.93–2.15)	1.52 (1.12–2.05)*	0.65 (0.48–0.87)*	0.64 (0.43–0.95)*
15	1.02 (0.98–1.06)	1.01 (0.98–1.04)	1.00 (0.97–1.03)	1.00 (0.96–1.04)	0–15	1.62 (1.04–2.51)*	1.62 (1.18–2.23)*	0.62 (0.45–0.83)*	0.60 (0.41–0.88)*
20	1.00 (0.96–1.05)	0.99 (0.96–1.03)	1.02 (0.98–1.06)	1.02 (0.97–1.08)	0–20	1.72 (1.10–2.67)*	1.63 (1.19–2.22)*	0.64 (0.48–0.87)*	0.64 (0.44–0.94)*
25	0.97 (0.93–1.00)	0.98 (0.95–1.01)	1.03 (1.00–1.06)	1.04 (1.00–1.08)	0–25	1.58 (1.02–2.45)*	1.51 (1.11–2.04)*	0.72 (0.54–0.97)*	0.76 (0.51–1.13)
30	0.92 (0.82–1.03)	0.96 (0.88–1.04)	1.03 (0.94–1.12)	1.03 (0.91–1.17)	0–30	1.15 (0.83–1.60)	1.26 (1.01–1.57)*	0.83 (0.68–1.01)	0.91 (0.71–1.18)

* *p*<0.05

## Discussion

It was reported that relative humidity had a significant inverse correlation with the prevalence of preeclampsia (*p* < 0.001) in Korea [11]. It was also reported that exposure to PM_2.5_, PM10, NO_2_ and O_3_ were risk factors for preeclampsia in the first and second trimesters of pregnancy, while at high levels, SO_2_ and CO were risk factors for preeclampsia in the second trimester in Hebei Province, China [12]. Therefore, the relative humidity and air pollution were considered in the DLNM model.

In the present study, a significant difference was found when the daily number of preeclampsia hospital admissions was divided into two subgroups (one called the cold-day subgroup, and the other called the hot-day subgroup based on the median of temperature). The daily number of preeclampsia hospital admissions was significantly larger on cold days than on hot days. At the same time, the temperature lag effects on the daily number of preeclampsia hospital admissions were studied using the DLNM model when considering the short-term effects. The curves of the relative risk on the daily number of preeclampsia hospital admissions were similar based on all three kinds of temperature (the daily mean temperature, daily minimum temperature and daily maximum temperature). The curves increased from a relative risk less than 1.0 to the peak, and then decreased from the peak to 1.0, when the temperature was lower than the reference temperature. At the same time, when the temperature was higher than the reference temperature, the relative risk decreased from 1.0 to the bottom, and then increased from the bottom to the peak.

To our knowledge, the association between the daily temperature and daily number of preeclampsia hospital admissions had rarely been studied. Most previous studies used seasonal or monthly temperature data, and focused on the long-term effects. Only one study used the daily maximum temperature, however there was no significant direct correlation between the number of preeclampsia cases and the daily maximum temperature in semiarid area [6]. In contrast to most previous studies, in this study, preeclampsia risk was considered based on the daily number of preeclampsia hospital admissions. In this way, it was more conducive to study the daily temperature effect on preeclampsia in the short-term.

We found that there was a significant difference between the daily number of preeclampsia hospital admissions in the low-temperature subgroup and the high-temperature subgroup using the median of temperature as threshold. Most previous studies found that preeclampsia risk was increased in women who conceived during the warm months and delivered during the cold months [3–5]. Preeclampsia occurred 20 weeks after conception, and this period was closer to the delivery period. This might partly explain why the daily number of preeclampsia hospital admissions at low temperatures was larger than that at high temperatures.

The daily number of preeclampsia hospital admissions time series indicated a weak correlation with the three daily temperature time series. Compared with the median temperature, a high temperature was a protective factor. There might be two reasons. One reason was that high temperature in Nanjing was not too extreme; another reason was that people reduced their outdoor activities on high-temperature days.

The curves of the relative risk on the daily number of preeclampsia hospital admissions were similar based on all three kinds of temperature (the daily mean temperature, daily minimum temperature and daily maximum temperature). First, the curves increased from the relative risk less than 1.0 to the peak. It was difficult to physiologically explain why the daily temperature was proportional to the preeclampsia relative risk when the daily temperature was very low. One possible explanation was human activity; when the weather was extremely cold, pregnant women reduced their outdoor time and remained in doors to keep warm. Then the relative risk curves decreased from the peak to 1.0 when the temperature was lower than the reference temperature. Although the mechanisms of the association between temperature and preeclampsia risk are not clear, some hypotheses have been proposed to explain the association between cold temperatures and preeclampsia risk. Kimura et al. [13] found that the pulsatility index of uterine artery blood flow significantly increased with cold exposure from 1.14 to 1.52 in patients with preeclampsia, whereas it increased from 0.95 to 1.25 in normal controls. Woisetschlager et al. [14] found that during the cold pressor test, systolic and diastolic blood pressure increased significantly and were more pronounced in women developing preeclampsia than in healthy pregnant women. At the same time, when the temperature was higher than the reference temperature, the relative risk first decreased and then increased with increasing of temperature. This result suggested that a high temperature was a protective factor preeclampsia risk; however, extremely high temperatures still led to a high relative risk.

When the temperature is low, it is recommended that pregnant women take measures to stay warm, pay attention to their blood pressure in the next 20 days, and go to the hospital when their blood pressure rises. At the same time, the government should increase publicity on the impact of low temperatures on preeclampsia, especially the lag effect of low temperatures on preeclampsia. All of the above could be used to help pregnant women and the government reduce the preeclampsia risk in Nanjing or other cities with similar climate conditions.

Our study had two strengths. First, the exposure–response association between temperature and preeclampsia risk was not studied on the short-term in previous studies. In this study, we first showed the exposure–response association between temperature and preeclampsia risk in a short-period of time. Second, the mean daily temperature, minimum temperature and maximum temperature were all studied, and all three kinds of temperature showed consistency.

Our study also had two limitations. First, we used ambient temperature, and when extreme hot or cold weather occurred, pregnant women usually remained in their houses; therefore, indoor temperature should be considered. Second, due to patient privacy, socioeconomic status and medical condition were not considered in this study.

## Conclusion

Our results showed that the daily number of preeclampsia hospital admissions was significantly larger at low daily temperatures than in high daily temperatures. The daily number of preeclampsia hospital admissions and daily ambient temperatures had a weak negative correlation in significant. The lag effect of low temperatures on preeclampsia was demonstrated to be a significant risk factor. These results could be used to help pregnant women and the government reduce preeclampsia risk. 

## Supplementary Information

**Additional file 1: Supplemental Material A. Table S1** The cumulative effects estimated for different temperatures (25th percentile and 75th percentile) at different lag days with the reference temperature. **Table S2** Model parameter selection for different lag days. **Supplemental Material B. Table S3** Model parameter selection for different degrees of freedom of RH, SO_2_, NO_2_, CO, O_3_, PM_10_ and PM_2.5_. **Supplemental Material C. Table S4** The total cumulative effects of relative risk for different degree of freedom of RH, SO_2_, NO_2_, CO, O_3_, PM_10_ and PM_2.5_ with temperature (25th percentile). **Table S5** The total cumulative effects of relative risk for different degree of freedom of RH, SO_2_, NO_2_, CO, O_3_, PM_10_ and PM_2.5_ with temperature (75th percentile).

## Data Availability

The datasets used and/or analysed during the current study available from the corresponding author upon reasonable request.

## References

[1] Ananth CV, Keyes KM, Wapner RJ (2013). Preeclampsia rates in the United States, 1980–2010: age-period-cohort analysis. BMJ.

[2] Mol BWJ, Roberts CT, Thangaratinam S, Magee LA, de Groot CJM, Hofmeyr GJ (2016). Preeclampsia. Lancet.

[3] Beltran AJ, Wu J, Laurent O (2013). Associations of meteorology with adverse pregnancy outcomes: a systematic review of preeclampsia, preterm birth and birth weight. Int J Environ Res Public Health.

[4] Auger N, Siemiatycki J, Bilodeau-Bertrand M, Healy-Profitós J, Kosatsky T (2017). Ambient temperature and risk of preeclampsia: biased association?. Paediatr Perinat Epidemiol.

[5] Xiong T, Chen P, Mu Y, Li X, Di B, Li J, Qu Y, Tang J, Liang J, Mu D (2020). Association between ambient temperature and hypertensive disorders in pregnancy in China. Nat Commun.

[6] Yackerson NS, Piura B, Friger M (2007). The influence of weather state on the incidence of preeclampsia and placental abruption in semi-arid areas. Clin Exp Obstet Gynecol.

[7] Basu R (2017). Temperature and preeclampsia: is the association valid?. Paediatr Perinat Epidemiol.

[8] Gasparrini A, Armstrong B, Kenward MG (2010). Distributed lag non-linear models. Stat Med.

[9] Gasparrini A (2011). Distributed Lag Linear and Non-Linear Models in R: The Package dlnm. J Stat Softw.

[10] Gasparrini A (2014). Modeling exposure-lag-response associations with distributed lag non-linear models. Stat Med.

[11] Cho GJ (2017). Effect of relative humidity on preeclampsia. Clin Exp Obstet Gynecol.

[12] Jia L, Liu Q, Hou H (2020). Association of ambient air pollution with risk of preeclampsia during pregnancy: a retrospective cohort study. BMC Public Health.

[13] Kimura Y, Okamura K, Watanabe T, Takahashi T, Haga I, Yajima A (1998). The Effect of cold stress on uterine artery blood flow velocity waveforms in late pregnant women with and without preeclampsia. Tohoku J Exp Med.

[14] Woisetschläger C, Waldenhofer U, Bur A, Herkner H, Kiss H, Binder M, Laggner AN, Hirschl MM (2000). Increased blood pressure response to the cold pressor test in pregnant women developing preeclampsia. J Hypertens.

