# Supplementary material for: Short-term effects of ambient temperature on the risk of preeclampsia in Nanjing, China: a time-series analysis
Source: BMC Pregnancy Childbirth. 2022 Jul 4;22:539. doi: 10.1186/s12884-022-04859-w (PMC9252039; doi:10.1186/s12884-022-04859-w)
Supplement: Supplementary file 1 — Additional file 1: Supplemental Material A. Table S1 The cumulative effects estimated for different temperatures (25th percentile and 75th percentile) at different lag days with the reference temperature. Table S2 Model parameter selection for different lag days. Supplemental Material B. Table S3 Model parameter selection for different degrees of freedom of RH, SO2, NO2, CO, O3, PM10 and PM2.5. Supplemental Material C. Table S4 The total cumulative effects of relative risk for different degree of freedom of RH, SO2, NO2, CO, O3, PM10 and PM2.5 with temperature (25th percentile). Table S5 The total cumulative effects of relative risk for different degree of freedom of RH, SO2, NO2, CO, O3, PM10 and PM2.5 with temperature (75th percentile). [file 12884_2022_4859_MOESM1_ESM.docx]

**Supplemental Material**

**Supplemental Material A**

Determination of the maximum lag days

We determined the maximum lag days according to the following two criteria. The first was to ensure that the cumulative relative risk from 0 days to the maximum lag days was statistically significant. The second was that the Bayesian information criterion (BIC) [10] value was small. We assumed the degrees of freedom of RH, SO_2_, NO_2_, CO, O_3_, PM_10_ and PM_2.5_ were 3. Considering that a cubic polynomial was used to fit the lag days, the minimum lag days was 3 days.

Table S1 shown the cumulative relative risk from a lag of 3 days to a lag of 30 days at the 25^th^ percentile and 75^th^ percentile. The cumulative effects of mean temperature from 3 lag days to 30 lag days at the 75^th^ percentile were statistically significant; the cumulative effects of minimum temperature from 0 lag days to 9 lag days at the 25^th^ percentile were statistically significant; and the cumulative effects of maximum temperature from 0 lag days to 30 lag days at the 25^th^ percentile were statistically significant.

Table S2 shown that the BIC value. The minimum BIC value of mean temperature was 2642.1 when the lag days were 30 days, ranging from 3 days to 30 days; the minimum BIC value on minimum temperature was 2736.4 when the lag days were 6 days, ranging from 3 days to 9 days and the minimum BIC value on maximum temperature was 2645.5 when the lag days were 30 days, ranging from 3 days to 30 days

Therefore, the maximum lag days of the mean temperature were 30 days; the maximum lag days of minimum temperature were 6 days and the maximum lag days of maximum temperature were 30 days.

Table S1. The cumulative effects estimated for different temperatures (25th percentile and 75th percentile) at different lag days with the reference temperature

|  | Mean temperature | | Minimum temperature | | Maximum temperature | |
| --- | --- | --- | --- | --- | --- | --- |
| lag | 25^th^ percentile | 75^th^ percentile | 25^th^ percentile | 75^th^ percentile | 25^th^ percentile | 75^th^ percentile |
| 0-3 | 1.23(1.02-1.48)* | 0.87(0.74-1.01) | 1.25(1.01-1.54)* | 0.88(0.77-1.01) | 1.20(1.00-1.44)* | 0.90(0.76-1.06) |
| 0-4 | 1.26(1.04-1.52)* | 0.85(0.73-1.00)* | 1.26(1.01-1.55)* | 0.89(0.77-1.02) | 1.23(1.02-1.48)* | 0.88(0.74-1.05) |
| 0-5 | 1.25(1.03-1.51)* | 0.86(0.73-1.01) | 1.26(1.01-1.56)* | 0.88(0.76-1.01) | 1.24(1.02-1.50)* | 0.87(0.73-1.04) |
| 0-6 | 1.26(1.04-1.53)* | 0.85(0.72-1.00)* | 1.26(1.01-1.56)* | 0.88(0.76-1.01) | 1.28(1.05-1.55)* | 0.86(0.72-1.02) |
| 0-7 | 1.27(1.05-1.55)* | 0.85(0.72-0.99)* | 1.26(1.01-1.57)* | 0.87(0.76-1.01) | 1.29(1.06-1.57)* | 0.85(0.71-1.01) |
| 0-8 | 1.25(1.03-1.52)* | 0.85(0.72-1.00)* | 1.25(1.01-1.56)* | 0.87(0.76-1.01) | 1.26(1.03-1.53)* | 0.85(0.71-1.02) |
| 0-9 | 1.25(1.02-1.52)* | 0.85(0.72-1.00)* | 1.25(1.00-1.56)* | 0.88(0.76-1.02) | 1.25(1.02-1.52)* | 0.84(0.70-1.01) |
| 0-10 | 1.24(1.02-1.51)* | 0.85(0.72-1.00)* | 1.23(0.99-1.54) | 0.88(0.76-1.02) | 1.26(1.03-1.54)* | 0.83(0.69-1.00)* |
| 0-11 | 1.23(1.01-1.51)* | 0.85(0.72-1.00) | 1.22(0.98-1.53) | 0.89(0.76-1.03) | 1.27(1.03-1.55)* | 0.83(0.69-0.99)* |
| 0-12 | 1.25(1.02-1.52)* | 0.85(0.72-1.00) | 1.23(0.98-1.54) | 0.89(0.77-1.04) | 1.28(1.04-1.57)* | 0.83(0.69-1.00)* |
| 0-13 | 1.26(1.03-1.54)* | 0.86(0.73-1.02) | 1.23(0.98-1.54) | 0.90(0.78-1.05) | 1.30(1.06-1.60)* | 0.83(0.69-1.00)* |
| 0-14 | 1.24(1.01-1.52)* | 0.87(0.74-1.03) | 1.21(0.96-1.51) | 0.91(0.78-1.06) | 1.29(1.05-1.58)* | 0.84(0.70-1.01) |
| 0-15 | 1.23(1.01-1.51)* | 0.88(0.74-1.03) | 1.20(0.96-1.51) | 0.91(0.78-1.06) | 1.27(1.03-1.56)* | 0.85(0.71-1.03) |
| 0-16 | 1.23(1.01-1.51)* | 0.88(0.74-1.04) | 1.21(0.96-1.51) | 0.91(0.78-1.06) | 1.27(1.03-1.56)* | 0.86(0.72-1.04) |
| 0-17 | 1.23(1.01-1.51)* | 0.88(0.74-1.04) | 1.21(0.97-1.52) | 0.91(0.78-1.06) | 1.26(1.03-1.55)* | 0.86(0.72-1.04) |
| 0-18 | 1.23(1.01-1.51)* | 0.88(0.74-1.04) | 1.21(0.97-1.52) | 0.91(0.78-1.06) | 1.26(1.03-1.55)* | 0.86(0.71-1.04) |
| 0-19 | 1.23(1.00-1.51)* | 0.88(0.74-1.04) | 1.21(0.96-1.52) | 0.91(0.78-1.07) | 1.26(1.02-1.55)* | 0.87(0.72-1.05) |
| 0-20 | 1.22(1.00-1.50) | 0.88(0.74-1.05) | 1.20(0.96-1.51) | 0.92(0.78-1.07) | 1.25(1.02-1.55)* | 0.87(0.72-1.06) |
| 0-21 | 1.22(0.99-1.49) | 0.88(0.74-1.05) | 1.20(0.95-1.50) | 0.91(0.78-1.06) | 1.25(1.01-1.54)* | 0.87(0.72-1.06) |
| 0-22 | 1.22(0.96-1.50) | 0.86(0.71-1.03) | 1.20(0.96-1.51) | 0.89(0.76-1.05) | 1.26(1.02-1.55)* | 0.86(0.71-1.05) |
| 0-23 | 1.21(0.96-1.50) | 0.89(0.72-1.01) | 1.20(0.96-1.51) | 0.88(0.75-1.03) | 1.26(1.02-1.56)* | 0.85(0.70-1.03) |
| 0-24 | 1.22(1.00-1.40) | 0.85(0.71-1.01) | 1.20(0.96-1.50) | 0.88(0.75-1.04) | 1.26(1.02-1.55)* | 0.84(0.70-1.02) |
| 0-25 | 1.21(0.99-1.49) | 0.85(0.71-1.01) | 1.19(0.94-1.49) | 0.89(0.76-1.04) | 1.26(1.02-1.56)* | 0.84(0.69-1.01) |
| 0-26 | 1.21(0.99-1.49) | 0.84(0.71-1.01) | 1.18(0.94-1.48) | 0.89(0.76-1.04) | 1.26(1.02-1.56)* | 0.83(0.69-1.01) |
| 0-27 | 1.22(0.99-1.49) | 0.84(0.70-1.00) | 1.18(0.94-1.48) | 0.88(0.75-1.04) | 1.27(1.03-1.58)* | 0.82(0.68-1.00) |
| 0-28 | 1.21(0.98-1.48) | 0.83(0.69-0.99)* | 1.16(0.92-1.46) | 0.88(0.74-1.03) | 1.27(1.02-1.58)* | 0.82(0.67-1.00)* |
| 0-29 | 1.20(0.98-1.48) | 0.83(0.69-0.99)* | 1.16(0.92-1.47) | 0.87(0.74-1.02) | 1.27(1.02-1.58)* | 0.82(0.67-1.00)* |
| 0-30 | 1.20(0.97-1.48) | 0.83(0.69-1.00)* | 1.16(0.92-1.47) | 0.87(0.73-1.02) | 1.26(1.01-1.57)* | 0.83(0.68-1.01) |

* *p*<0.05

Table S2. Model parameter selection for different lag days

| Lag days | BIC value  (mean temperature) | BIC value  (minimum temperature) | BIC value  (maximum temperature) |
| --- | --- | --- | --- |
| 3 | 2753.8 | 2743.2 | 2742.8 |
| 4 | 2746.5 | 2745.3 | 2745.0 |
| 5 | 2745.4 | 2742.6 | 2743.5 |
| 6 | 2744.9 | 2736.4 | 2736.8 |
| 7 | 2742.0 | 2737.6 | 2732.4 |
| 8 | 2734.7 | 2741.3 | 2727.0 |
| 9 | 2733.4 | 2738.3 | 2721.4 |
| 10 | 2732.2 | 2731.8 | 2725.9 |
| 11 | 2725.6 | 2726.4 | 2723.6 |
| 12 | 2714.3 | 2718.9 | 2717.1 |
| 13 | 2705.8 | 2714.3 | 2706.9 |
| 14 | 2697.7 | 2703.3 | 2695.1 |
| 15 | 2692.9 | 2698.5 | 2694.7 |
| 16 | 2686.3 | 2691.2 | 2686.7 |
| 17 | 2685.4 | 2691.6 | 2685.1 |
| 18 | 2684.7 | 2690.2 | 2684.2 |
| 19 | 2681.3 | 2686.2 | 2682.0 |
| 20 | 2680.0 | 2682.6 | 2680.4 |
| 21 | 2676.1 | 2679.1 | 2675.9 |
| 22 | 2670.3 | 2672.8 | 2672.1 |
| 23 | 2661.8 | 2664.7 | 2664.3 |
| 24 | 2659.5 | 2665.5 | 2655.8 |
| 25 | 2659.0 | 2664.6 | 2654.6 |
| 26 | 2658.2 | 2661.7 | 2656.0 |
| 27 | 2656.8 | 2658.0 | 2658.0 |
| 28 | 2651.4 | 2651.3 | 2654.2 |
| 29 | 2649.5 | 2650.1 | 2653.5 |
| 30 | 2642.1 | 2643.9 | 2645.5 |

**Supplemental Material B**

Table S3. Model parameter selection for different degrees of freedom of RH, SO_2_, NO_2_, CO, O_3_, PM_10_ and PM_2.5_

|  | BIC value of mean temperature  Lag 30 days | | | BIC value of minimum temperature  Lag 6 days | | | BIC value of maximum temperature  Lag 30 days | | |
| --- | --- | --- | --- | --- | --- | --- | --- | --- | --- |
| Degree of freedom | 3 | 4 | 5 | 3 | 4 | 5 | 3 | 4 | 5 |
| RH | 2642.1 | 2650.0 | 2659.0 | 2736.4 | 2744.9 | 2753.6 | 2645.5 | 2653.7 | 2662.7 |
| SO_2_ | 2642.1 | 2651.1 | 2660.5 | 2736.4 | 2745.5 | 2754.8 | 2645.5 | 2654.3 | 2663.7 |
| NO_2_ | 2642.1 | 2651.4 | 2660.0 | 2736.4 | 2745.9 | 2753.9 | 2645.5 | 2654.9 | 2663.3 |
| CO | 2642.1 | 2648.7 | 2655.0 | 2736.4 | 2744.2 | 2750.1 | 2645.5 | 2652.8 | 2659.4 |
| O_3_ | 2642.1 | 2649.4 | 2654.8 | 2736.4 | 2742.2 | 2747.5 | 2645.5 | 2652.1 | 2656.6 |
| PM_10_ | 2642.1 | 2650.0 | 2659.1 | 2736.4 | 2745.5 | 2754.7 | 2645.5 | 2653.9 | 2663.3 |
| PM_2.5_ | 2642.1 | 2649.8 | 2658.9 | 2736.4 | 2745.6 | 2754.9 | 2645.5 | 2653.9 | 2663.0 |

* *p*<0.05

**Supplemental Material C**

Table S4. The total cumulative effects of relative risk for different degree of freedom of RH, SO_2_, NO_2_, CO, O_3_, PM_10_ and PM_2.5_ with temperature (25th percentile)

|  | mean temperature  Lag = 0-30 | | minimum temperature  Lag = 0-6 | | maximum temperature  Lag = 0-30 | |
| --- | --- | --- | --- | --- | --- | --- |
| Degree of freedom | 3 | 4 | 3 | 4 | 3 | 4 |
| RH | 1.20(0.97-1.48) | 1.20(0.98-1.48) | 1.26(1.01-1.56)* | 1.26(1.01-1.56)* | 1.26(1.01-1.57)* | 1.26(1.01-1.57)* |
| SO_2_ | 1.20(0.97-1.48) | 1.20(0.98-1,49) | 1.26(1.01-1.56)* | 1.26(1.01-1.56)* | 1.26(1.01-1.57)* | 1.26(1.01-1.58)* |
| NO_2_ | 1.20(0.97-1.48) | 1.20(0.97-1.48) | 1.26(1.01-1.56)* | 1.26(1.01-1.56)* | 1.26(1.01-1.57)* | 1.26(1.01-1.57)* |
| CO | 1.20(0.97-1.48) | 1.19(0.96-1.47) | 1.26(1.01-1.56)* | 1.25(1.01-1.55)* | 1.26(1.01-1.57)* | 1.25(1.00-1.56)* |
| O_3_ | 1.20(0.97-1.48) | 1.20(0.97-1.48) | 1.26(1.01-1.56)* | 1.26(1.01-1.56)* | 1.26(1.01-1.57)* | 1.25(1.01-1.56)* |
| PM_10_ | 1.20(0.97-1.48) | 1.21(0.98-1,49) | 1.26(1.01-1.56)* | 1.26(1.01-1.56)* | 1.26(1.01-1.57)* | 1.27(1.02-1.58)* |
| PM_2.5_ | 1.20(0.97-1.48) | 1.21(0.98-1.50) | 1.26(1.01-1.56)* | 1.26(1.01-1.57)* | 1.26(1.01-1.57)* | 1.27(1.02-1.59* |

Table S5. The total cumulative effects of relative risk for different degree of freedom of RH, SO_2_, NO_2_, CO, O_3_, PM_10_ and PM_2.5_ with temperature (75th percentile)

|  | mean temperature  Lag = 0-30 | | minimum temperature  Lag = 0-6 | | maximum temperature  Lag = 0-30 | |
| --- | --- | --- | --- | --- | --- | --- |
| Degree of freedom | 3 | 4 | 3 | 4 | 3 | 4 |
| RH | 0.83(0.69-1.00)* | 0.83(0.69-1.00)* | 0.88(0.76-1.01) | 0.87(0.76-1.01) | 0.83(0.68-1.01) | 0.83(0.68-1.01) |
| SO_2_ | 0.83(0.69-1.00)* | 0.83(0.69-0.99)* | 0.88(0.76-1.01) | 0.87(0.76-1.01) | 0.83(0.68-1.01) | 0.82(0.68-1.01) |
| NO_2_ | 0.83(0.69-1.00)* | 0.83(0.69-1.00)* | 0.88(0.76-1.01) | 0.87(0.76-1.01) | 0.83(0.68-1.01) | 0.83(0.68-1.01) |
| CO | 0.83(0.69-1.00)* | 0.83(0.69-1.00) | 0.88(0.76-1.01) | 0.88(0.76-1.01) | 0.83(0.68-1.01) | 0.83(0.68-1.02) |
| O_3_ | 0.83(0.69-1.00)* | 0.83(0.69-0.99)* | 0.88(0.76-1.01) | 0.88(0.76-1.01) | 0.83(0.68-1.01) | 0.83(0.68-1.01) |
| PM_10_ | 0.83(0.69-1.00)* | 0.83(0.69-1.00)* | 0.88(0.76-1.01) | 0.88(0.76-1.01) | 0.83(0.68-1.01) | 0.83(0.68-1.01) |
| PM_2.5_ | 0.83(0.69-1.00)* | 0.82(0.69-0.99)* | 0.88(0.76-1.01) | 0.87(0.76-1.01) | 0.83(0.68-1.01) | 0.82(0.67-1.01) |

* *p*<0.05
